# Predictors of caspofungin exposure-response relationship: a retrospective cohort study on therapeutic drug monitoring parameters

**DOI:** 10.3389/fphar.2025.1620179

**Published:** 2025-07-28

**Authors:** Mingwei Meng, Ruhua Wei, Yating Lu, Wen Cao, Kai Mo, Zheng Huang, Yane Qin, Xiaobu Lan

**Affiliations:** ^1^Department of Pharmacy, The Fifth Affiliated Hospital of Guangxi Medical University & The First People’s Hospital of Nanning, Nanning, China; ^2^Department of Pharmacy, Guangxi International Zhuang Medicine Hospital, Nanning, China

**Keywords:** caspofungin, trough concentration, antifungal, efficacy, therapeutic drug monitoring

## Abstract

**Introduction:**

This study aimed to characterize the exposure-response relationship of caspofungin through analysis of trough serum concentration (Cmin) associated with clinical efficacy, while identifying clinically determinants of Cmin.

**Methods:**

A single-center retrospective cohort study collected therapeutic drug monitoring data from 83 hospitalized patients receiving caspofungin therapy between January 2022 and January 2025. Spearman correlation analysis and the receiver operating characteristic (ROC) curve were employed to evaluate associations between trough serum concentrations and clinical endpoints, with concomitant assessment of binary logistic regression analysis of determinants affecting interindividual exposure variability.

**Results:**

A total of 83 eligible cases were included, with 51 cases in the effective treatment group and 32 cases in the ineffective group. The median caspofungin Cmin in the effective group was 5.62 [4.54, 7.0] μg/mL, while that in the ineffective group was 2.43 [1.61, 3.03] μg/mL. Spearman correlation analysis revealed a positive correlation between caspofungin Cmin and clinical efficacy, with r = 0.8 and *p* < 0.01. The ROC curve for caspofungin Cmin was 0.974, with a maximum Youden index of 0.816, corresponding to a cutoff value of 3.58 μg/mL, a sensitivity of 94.1%, and a specificity of 87.5%. Binary logistic regression analysis showed that patient body weight [OR = 0.839 (0.765∼0.921), *p* < 0.001] was an influencing factor of caspofungin Cmin.

**Conclusion:**

Caspofungin trough serum concentration is correlated with its efficacy. Maintaining a trough concentration of caspofungin greater than 3.58 μg/mL within the dosing interval may achieve better clinical efficacy. Additionally, patient body weight should be considered when optimizing the dosing regimen due to its impact on drug concentration.

## 1 Introduction

Caspofungin was the first echinocandin antifungal drug to be licensed and has wided used for treatment of *candida* correlative bloodstream infections and other infections, as well as empirical antifungal therapy for patients with prolonged fever and neutropenia, and invasive aspergillus infections that are resistant or intolerant to other antifungal drugs (such as amphotericin B, liposomal amphotericin B, or itraconazole) (Lewis, 2011). Caspofungin exhibits concentration-dependent fungicidal activity against *Candida* spp., characterized by a prolonged post-antifungal effect. The pharmacokinetic/pharmacodynamic (PK/PD) targets of caspofungin are defined by the AUC/MIC (area under the concentration-time curve to minimum inhibitory concentration ratio) and Cmax/MEC (peak concentration to minimum effective concentration ratio) (Morris and Villmann, 2006). Integrated pharmacodynamic analyses indicate that maintaining caspofungin’s AUC_0-24_/MIC ratio between 450–1,185 or Cmax/MEC between 10–20 is required to achieve optimal therapeutic efficacy against *Candida* spp. and *Aspergillus* spp (Antachopoulos et al., 2007; Andes et al., 2010; Pound et al., 2010).

However, accurate AUC determination necessitates frequent blood sampling, which poses challenges to patient adherence and practical implementation in clinical settings. Additionally, the extensive plasma protein binding of caspofungin results in polyphasic pharmacokinetics, and the absence of a well-defined Tmax (time to peak concentration) range introduces uncertainty in determining the optimal sampling window for Cmax (peak concentration) measurement. Furthermore, in critically ill patients, empiric antifungal therapy is often initiated guided by local institutional epidemiology, including prevalent fungal species and their resistance patterns, prior to definitive pathogen identification. Given the unavailability of definitive MIC data prior to pathogen identification, therapeutic drug monitoring of caspofungin may utilize trough concentration (Cmin) as a pragmatic surrogate marker when AUC or Cmax based exposure assessments prove clinically impractical. Early *in vitro* PD studies established caspofungin’s MIC_90_ range against *Candida albicans* as 0.12–1 μg/mL (Ostrosky-Zeichner et al., 2003; Pfaller et al., 2008a; Pfaller et al., 2008b), proposing that a Cmin ≥1 μg/mL represents a potential therapeutic target for invasive candidiasis. Nevertheless, clinical validation through rigorous *in vivo* PD studies remains unavailable to substantiate this hypothesis.

This study used a retrospective analysis method enrolled 83 patients undergoing caspofungin treatment, exploring the correlation between caspofungin Cmin and clinical efficacy, while identifying key determinants of interindividual variability in drug exposure, to provide a reference for individualized caspofungin dosing regimens.

## 2 Materials and methods

### 2.1 Study design and patient data

A retrospective analysis design study was adopted. All the patients included in this study were Chinese. Patients hospitalized in the First People’s Hospital of Nanning from December 2022 to February 2025 were eligible for inclusion if they met the following criteria: 1) over 18 years of age; 2) intravenous infusion of caspofungin for more than 48 h; 3) underwent trough serum concentration monitoring 48 h after the start of caspofungin treatment. Exclusion criteria included: 1) patients with missing baseline data; 2) patients allergic to caspofungin. The study was approved by the ethics committee of the First People’s Hospital of Nanning.

### 2.2 Dosing regimen

The dose of caspofungin acetate for injection (Merck Sharp & Dohme Ltd., approval number: H20171218) was an initial loading dose of 70 mg with determination by the physicians, and tnen followed by a maintenance dose of 50 mg once daily starting from the second day, administered as an intravenous infusion over 1 h.

### 2.3 Measurement of caspofungin trough concentrations

The blood tests analyzed in this study were performed according to standard medical protocols. Blood samples were obtained to determine the caspofungin Cmin, which collected at least 48 h after the start of intravenous infusion and 30 min prior to the next dose. The serum concentration of caspofungin was measured using the automatic two-dimensional high-performance liquid chromatography system (FLC-2801 system, Demeter Instrument Co. Ltd., Changsha, China). Specifically, the calibration range for caspofungin serum concentrations is 0.54–25.87 μg/mL. And the precision and accuracy are both within 15%.

### 2.4 Data collection

The data collection process was conducted utilizing the hospital information management system, with basic patient characteristics include gender, age, body weight, duration of caspofungin treatment, concurrent antibiotic usage, infection site, microbiological results and various laboratory data (including blood routine examination results, albumin concentration, inflammatory parameters and renal and liver function). Creatinine clearance (CrCl) was calculated using the Cockcroft-Gault formula:

$$\text{CrCl }\left(\text{mL}/\min\right)\;\left(\text{for males}\right)=\left(140-\text{age}\right)\times \text{body weight}\left(\text{kg}\right)/\left[0.818\times \text{serum creatinine }\left(\mu \text{mol}/L\right)\right];\text{ CrCl }\left(\text{for females}\right)=\text{CrCl }\left(\text{male}\right)\times 0.85.$$

### 2.5 Evaluation of clinical treatment effect

The clinical treatment effect was evaluated according to the “Technical Guidelines for Antimicrobial Clinical Trials” issued by the former China Food and Drug Administration, assessing three aspects: clinical efficacy, microbiological efficacy, and overall efficacy. The explicit criteria defining treatment effectiveness as follows: (1) Effective treatment: Clinical cure at the end of treatment visit with bacterial eradication or presumed eradication. (2) Ineffective treatment: Clinical failure at the end of treatment visit and/or persistence of the baseline bacterial pathogen (Writing Group of Guidance for Clinical Trials of Anti-bacterial Drugs, 2014).

### 2.6 Statistical methods

Data processing was performed using SPSS version 23.0 software. Normally distributed continuous variables were described as mean ± standard deviation (x ± s), with comparisons between groups using independent sample t-tests. Non-normally distributed continuous variables were described as median [interquartile range, IQR], with comparisons between groups using the Mann-Whitney U test. Categorical variables were described as number of cases (N) or percentage (%), with comparisons between groups using the chi-square test. Spearman correlation analysis was used to analyze the correlation between Cmin and clinical efficacy. The receiver operating characteristic (ROC) curve was used to assess the predictive value of Cmin for clinical efficacy. The sensitivity, specificity, and area under the ROC curve were calculated, severally. The cutoff value was determined using the Youden index. Binary linear regression analysis was used to analyze the influencing factors of Cmin. A *p* value of <0.05 was considered statistically significant.

## 3 Results

### 3.1 Characteristics of patients

Base on the inclusion and exclusion criteria, a total of 83 patients were enrolled in the study, their demographics were summarized in Table 1. The treatment outcomes were evaluated based on the criteria outlined in “Section 2.5”. Ultimately, 51 patients were regarded as have responded effectively to treatment, while the other 32 patients were regarded as ineffective. Specifically, significant differences were found in body weight and platelet count between the effective and ineffective groups (*p* < 0.05). The details are shown in Table 1.

**TABLE 1:** Comparison of clinical characteristics of patients between two groups.

Parameters	The effective group (n = 51)	The ineffective group (n = 32)	Value	P-value
Sex (male), N (%)	29 (56.86)	23 (71.88)	2.496	0.114
Age (year)	70.96 ± 14.25	64.75 ± 17.1	1.788	0.077
Body weight (kg)	55 [46.75, 60.5]	62.5 [51, 67]	−2.468	0.014
WBC (×10^9^*L^−1^)	9.95 [6.49, 13.53]	7.93 [6.01, 10.95]	−1.455	0.146
PLT (×10^9^*L^−1^)	218.53 ± 134.02	141.47 ± 122.71	2.63	0.01
ALT (U* L^−1^)	17.3 [9, 27.7]	19.1 [7.53, 33.75]	−0.103	0.918
AST (U* L^−1^)	30.6 [20.6, 42.25]	33.1 [15.45, 49.18]	−0.257	0.797
ALP(U* L^−1^)	76.0 [60.2, 128.55]	92.0 [63.65, 124.75]	−0.655	0.513
TB (μmoL*L^−1)^	10.1 [7.15, 16.15]	12 [7.28, 25.4]	−0.571	0.568
ALB (g*L^−1^)	30.44 ± 3.93	30.26 ± 5.04	0.189	0.851
SCR (μmol*L^−1^)	87 [63, 180]	127.5 [73, 302]	−0.95	0.342
CrCl (mL*min^−1^)	44.65 [22.4, 71.25]	42.06 [15.95, 95.36]	−0.327	0.743
Administration period (d)	15.04 ± 4.55	14.25 ± 5.47	0.711	0.479
Infection site, N (%)				
Pulmonary infection	41 (80.39)	21 (65.63)	2.269	0.123
Bloodstream infection	7 (13.73)	6 (18.75)	0.376	0.54
Abdominal infection	3 (5.88)	5 (15.62)	1.17	0.279
Organism, N (%)				
*Candida albicans*	19 (37.26)	8 (25.0)	1.345	0.246
*Candida parapsilosis*	3 (5.88)	6 (18.75)	2.168	0.141
*Candida tropicalis*	13 (25.49)	7 (21.87)	0.141	0.708
*Candida glabrata*	9 (17.65)	9 (28.13)	1.271	0.26
*Candida krusei*	2 (3.92)	0	-	0.52
Empirical treatment	5 (9.8)	2 (6.25)	0.026	0.872

Data are expressed as numbers (%) for categorical variables, mean ± SD or median [IQR] for continuous variables. WBC: white blood cell count; PLT: platelet; ALT: alanine aminotransferase; AST: aspartate aminotransferase; ALP: alkaline phosphatase; TB: total bilirubin; ALB: albumin; SCR: serum creatinine; CrCl: Creatinine clearance.

### 3.2 Analysis of caspofungin serum concentration

**FIGURE 1:**
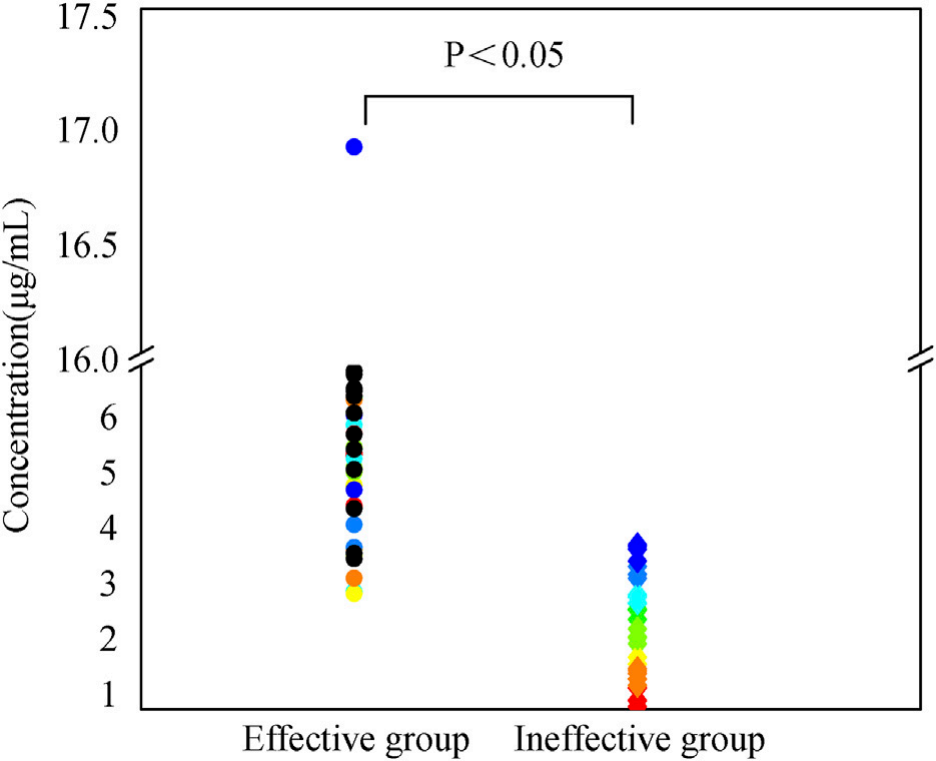
Caspofungin dosing regimen consisted of a 70 mg initial loading dose, followed by a 50 mg maintenance dose starting from day 2. After 2 days of treatment according to this regimen, trough serum concentrations were measured prior to the next dose administration. Patients were categorized into effective and ineffective groups based on clinical efficacy, microbiological efficacy, and overall efficacy. Trough serum concentrations were compared between the two groups. Continuous variables not normally distributed were described as median [IQR], with comparisons between groups using the Mann-Whitney U test.

In this study, the median caspofungin Cmin in the effective group was 5.62 [4.54, 7.0] μg/mL, while in the ineffective group was 2.43 [1.61, 3.03] μg/mL. The Cmin in the effective group was higher than that in the ineffective group, with a statistically significant difference (*p* < 0.05), as shown in Figure 1.

### 3.3 Analysis of the correlation between caspofungin Cmin and clinical efficacy

**FIGURE 2:**
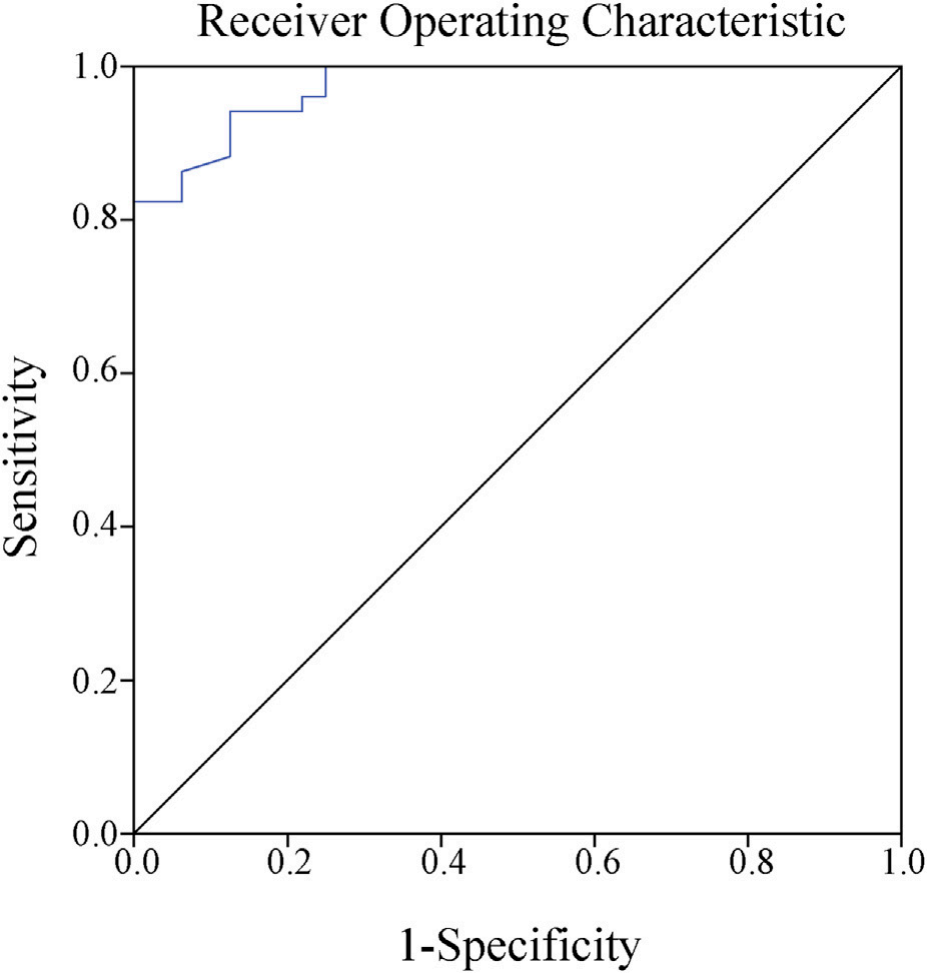
Receiver operating characteristic curve showed that the best cutoff point for caspofungin trough serum concentrations to predict the clinical efficacy was 3.58 μg/mL (sensitivity 94.1%, specificity 87.5%, Jorden index 0.816, area under the curve AUC 0.974).

The results of the Spearman correlation analysis revealed a positive correlation between caspofungin Cmin and clinical efficacy, with r = 0.8 and *p* < 0.01. The ROC curve was plotted to further evaluate the predictive value of caspofungin Cmin for clinical efficacy. The area under the ROC curve was 0.974, with a maximum Youden index of 0.816. The corresponding cutoff value was 3.58 μg/mL, with a sensitivity of 94.1% and a specificity of 87.5% (Figure 2). This result indicate that when the caspofungin Cmin maintain greater than 3.58 μg/mL, it is likely to achieve better clinical efficacy.

### 3.4 Analysis of influencing factors of caspofungin Cmin

**FIGURE 3:**
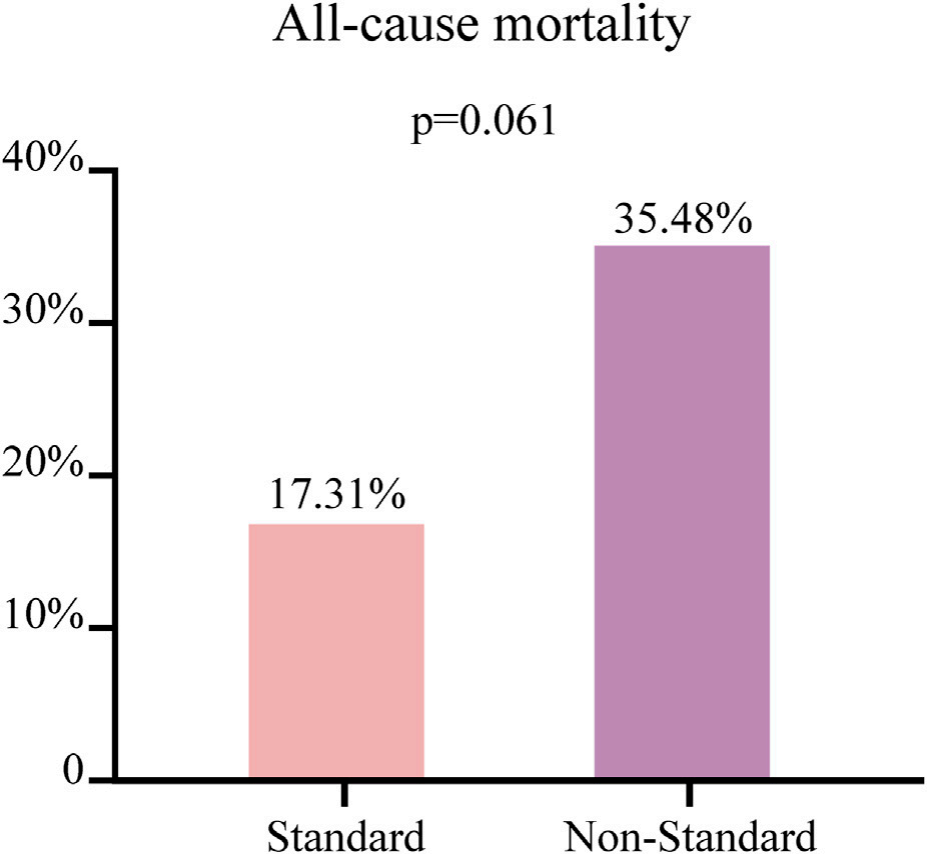
The patients with a concentration greater than 3.58 μg/mL classified as the standard group (n = 52) and a concentration less than 3.58 μg/mL classified as the non-standard group (n = 31). Comparison of the all-cause mortality between two groups of patients. Continuous variables not normally distributed were described as median [IQR], with comparisons between groups using the Mann-Whitney U test.

The 83 patients were grouped based on their Cmin values, with a concentration greater than 3.58 μg/mL classified as the standard group (n = 52) and a concentration less than 3.58 μg/mL classified as the non-standard group (n = 31), to further explored the influencing factors of caspofungin Cmin. Univariate analysis showed that there were significant differences in age and body weight between the two groups (*p* < 0.05), as shown in Table 2. The all-cause mortality was 17.31% (9/52) in the standard group and 35.48% (11/31) in the non-standard group. This value is lower in the standard group than in the non-standard group, although there was no statistical difference (*p* > 0.05), as shown in Figure 3.

**TABLE 2:** Results of univariate analysis of carpofungin Cmin with standard group and non-standard group.

Parameters	The standard group (n = 52)	The non-standard group (n = 31)	Value	P-value
Sex (male), N (%)	29 (55.77)	23 (74.19)	2.179	0.14
Age (year)	71.19 ± 14.26	64.16 ± 16.98	2.02	0.046
Body weight (kg)	54.47 ± 8.47	62.44 ± 5.36	−5.505	<0.01
ALT (U* L^−1^)	179 [9.05, 28.65]	17 [7.35, 32]	−0.603	0.547
AST (U* L^−1^)	32.95 [21.88, 45.83]	26.5 [15.1, 40.55]	−1.294	0.196
TB (μmoL*L^−1)^	11.15 [7.5, 17.98]	10.3 [6.05, 15.85]	−0.607	0.544
ALB (g*L^−1^)	30.55 ± 3.91	30.26 ± 5.04	0.476	0.635
SCR (μmol*L^−1^)	92 [63.75, 179.5]	103 [67.5, 332]	−0.386	0.699
CrCl (mL*min^−1^)	42.74 [20.32, 70.21]	49.22 [15.88, 101.71]	−0.904	0.366
Administration period (d)	15.06 ± 4.43	14.19 ± 5.64	0.775	0.441

Data are expressed as numbers (%) for categorical variables, mean ± SD or median [IQR] for continuous variables. ALT: alanine aminotransferase; AST: aspartate aminotransferase; TB: total bilirubin; ALB: albumin; SCR: serum creatinine; CrCl: Creatinine clearance.

Binary logistic regression analysis was conducted to identify the determinants of the target caspofungin Cmin. The following variables were included: age, body weight, albumin (ALB), serum creatinine (SCR), total bilirubin (TB), alkaline phosphatase (ALP), alanine aminotransferase (ALT), and aspartate aminotransferase (AST). The results are shown in Table 3. The body weight [OR = 0.839 (0.765∼0.921), *p* < 0.001] was an influencing factor for achieving the target caspofungin Cmin.

**TABLE 3:** The results of binary logistic regression of carpofungin Cmin.

Parameters	OR	95% confidence interval	P-value
Age	1.02	0.984–1.057	0.284
Body weight	0.839	0.765–0.921	<0.001
ALB	1.024	0.898–1.168	0.719
SCR	0.999	0.996–1.002	0.549
TB	1.003	0.992–1.014	0.581
ALP	1.001	0.995–1.006	0.756
ALT	1.014	0.979–1.050	0.449
AST	0.997	0.975–1.019	0.773

ALB: albumin; SCR: serum creatinine; TB: total bilirubin; ALP: alkaline phosphatase; ALT: alanine aminotransferase; AST: aspartate aminotransferase.

## 4 Discussion

While current evidence demonstrates a paucity of large-scale clinical trial data evidence to endorse routine therapeutic drug monitoring (TDM) implementation for echinocandin antifungals (micafungin, caspofungin, anidulafungin), emerging individualized drug therapy suggest that characterization of their exposure-response relationships through TDM and PK/PD target attainment analyses may provide a clinically actionable adjunct to contemporary precision antimicrobial stewardship paradigms.

Caspofungin has demonstrated satisfactory both *in vitro* and *in vivo* fungicidal activity against most *Candida* species, including *Candida albicans*, *Candida glabrata*, *Candida tropicalis*, *Candida dubliniensis*, and *Candida krusei*. Additionally, although it show a low activity against *Candida parapsilosis* and *Candida guilliermondii*, caspofungin has high MICs against these species (Ostrosky-Zeichner et al., 2003). Early *in vitro* antifungal activity studies have shown that the MIC_90_ range of caspofungin for *Candida albicans* is 0.12 μg/mL–1 μg/mL (Ostrosky-Zeichner et al., 2003; Pfaller et al., 2008a; Pfaller et al., 2008b). This research suggests that a plasma drug concentration of 1 μg/mL may be a potential effective target concentration for the treatment of *Candida* infections (Pfaller et al., 2008b). Furthermore, in a pharmacokinetic and pharmacodynamic study, for exploring the dose of caspofungin, healthy volunteers were given daily doses ranging from 15 to 70 mg for 14 consecutive days. The results showed that only the 70 mg group maintained plasma concentrations above 1 μg/mL throughout the study, while the 50 mg group maintained concentrations above the effective target concentration after 3 consecutive days of dosing. Further research found that an initial dose of 70 mg followed by a maintenance dose of 50 mg daily could ensure that plasma concentrations remained above the potential effective therapeutic concentration (Stone E. A. et al., 2002). This may be one of the reasons for routine TDM for caspofungin is not currently recommended, but there is still a lack of reliable clinical data to support this. In this study, antifungal susceptibility testing results were obtained for only a small subset of patients. These results revealed that the MIC_90_ range of caspofungin for *Candida albicans*, *Candida parapsilosis*, *Candida tropicalis*, *Candida glabrata*, *Candida krusei* were 0.03 μg/mL–0.5 μg/mL. Notably, our study revealed a new phenomenon that while 98.8% (82/83) of patients achieved trough serum concentrations exceeding the putative target threshold of 1 μg/mL, only 61.4% (51/83) attained composite clinical efficacy endpoints. The result suggest that a potential target plasma concentration of 1 μg/mL may not be applicable to all clinical patients.

We further analyzed the ROC curve and found that patients with a caspofungin Cmin greater than 3.58 μg/mL achieved better clinical efficacy. It is worth mentioning that this target value also takes into account other non-*Candida albicans* fungi. Our study revealed a higher treatment failure rate associated with non-*Candida albicans* species during caspofungin therapy. For instance, *Candida glabrata* was isolated from 17.65% (9/51) of patients in the effective group, versus 28.13% (9/32) in the ineffective group. Similarly, *Candida parapsilosis* was identified in 5.88% (3/51) of the effective group, compared to 18.75% (6/32) of the ineffective group, as shown in Table 1. Multiple studies of caspofungin population pharmacokinetic (PPK) models have shown that when the MIC of *Candida parapsilosis* is greater than 0.25 μg/mL, conventional dosing regimens may lead to treatment failure (Pérez-Pitarch et al., 2018; Niu et al., 2020; Li et al., 2021).

Usually, caspofungin is poorly bioavailable for oral use. Following intravenous infusion, it becomes highly protein-bound and distribute well into tissues, resulting in a rapid decline in plasma concentrations. In the liver, caspofungin undergoes spontaneous disintegration to an open-ring compound followed by peptide hydrolysis and n-acytylation into two inactive metabolites (Stone J. A. et al., 2002). These metabolites, along with a portion of unchanged caspofungin, are ultimately excreted via urine and feces (Stevens, 2012). Dose adjustment is also not required in patients with hepatic impairment or renal dysfunction (Morris and Villmann, 2006; Dowell et al., 2013). However, Sinnollareddy et al. (2015) found that caspofungin exhibits high pharmacokinetic variability and a considerable risk of low exposure in different populations, particularly in critically ill patients. These factors contribute to the suboptimal clinical efficacy of caspofungin. Changes in patient organ function, continuous renal replacement therapy, and extracorporeal membrane oxygenation are important factors that can alter the PK parameters of caspofungin (Nguyen et al., 2007; Smith et al., 2012; Muilwijk et al., 2014). In our enrolled 83 patients, there was significant individual variability in trough concentrations (4.66 ± 2.66 μg/mL) despite they are received the same dose of caspofungin. We further explored the factors influencing caspofungin trough concentrations. Binary logistic regression analysis revealed that body weight was correlated with caspofungin Cmin (*p* < 0.05), suggested that clinical dosing may adjusted based on the patient’s body weight. Because of the inverse relationship between drug concentration and volume of distribution, heavier patients could have lower caspofungin concentrations under the same treatment strategies. In previous studies on caspofungin PPK (Hall et al., 2013; Würthwein et al., 2013; van der Elst et al., 2017; Märtson et al., 2020), body weight was identified as a major covariate affecting caspofungin clearance (CL) and volume of distribution (Vd), which is consistent with our findings. Additionally, we stratified patients into two groups based on whether the caspofungin Cmin reached 3.58 μg/mL and compared their clinical outcomes during hospitalization. The results revealed that, despite the absence of statistical significance, the standard group demonstrated a numerically lower all-cause mortality rate, which might be attributed to the limited sample size of the study cohort.

In summary, our findings substantiate the imperative role of TDM in optimizing caspofungin’s precision pharmacotherapy. By systematically investigating the association between caspofungin Cmin and clinical efficacy, we implemented a streamlined sampling protocol that minimized both iatrogenic risks and healthcare costs associated with excessive blood collection in patients. Our results indicate that patients with a caspofungin Cmin greater than 3.58 μg/mL are more likely to achieve better treatment outcomes, which may provide a reference for individualized dosing of caspofungin.

This study has several constraints that warrant consideration: a) The limited cohort size that only 2.4% (2/83) of patients developed liver injury, precluding meaningful statistical analysis of concentration-toxicity relationships; b) The high prevalence of hypoalbuminemia in the study population introduced significant confounding effects, restricting covariate analysis of protein binding-mediated pharmacokinetic alterations and their therapeutic implications; c) The enrolled patients were all adults, and there was a lack of clinical data for pediatric patients.

## Data Availability

The original contributions presented in the study are included in the article/supplementary material, further inquiries can be directed to the corresponding author.
